# External validation of a model to predict recurrence-free and melanoma-specific survival for patients with melanoma after sentinel node biopsy

**DOI:** 10.1093/bjs/znaf037

**Published:** 2025-04-17

**Authors:** Robert C Stassen, Carolien C H M Maas, Stanley P Leong, Mohammed Kashani-Sabet, Richard L White, Barbara A Pockaj, Jonathan S Zager, Schlomo Schneebaum, John T Vetto, Eli Avisar, J Harrison Howard, Cristina O’Donoghue, Heidi Kosiorek, Alexander C J van Akkooi, Cornelis Verhoef, David van Klaveren, Dirk J Grünhagen, Roger Olofsson Bagge

**Affiliations:** Department of Surgical Oncology, Erasmus Medical Centre Cancer Institute, Rotterdam, The Netherlands; Department of Public Health, Erasmus University Medical Centre, Rotterdam, The Netherlands; Department of Surgery, California Pacific Medical Center and Research Institute, San Francisco, California, USA; Department of Surgery, California Pacific Medical Center and Research Institute, San Francisco, California, USA; Department of Surgery, Levine Cancer Institute, Carolinas Medical Center, Atrium Health, Charlotte, North Carolina, USA; Department of Surgery, Mayo Clinic, Phoenix, Arizona, USA; Department of Cutaneous Oncology, Moffitt Cancer Center, Tampa, Florida, USA; Department of Surgery, Tel-Aviv Sourasky Medical Center, Tel Aviv, Israel; Division of Surgical Oncology, Oregon Health & Science University, Portland, Oregon, USA; Department of Surgery, Division of Surgical Oncology at University of Miami Miller School of Medicine, Miami, Florida, USA; Department of Surgery, University of South Alabama, Mobile, Alabama, USA; Department of Surgery, Rush University Medical Center, Chicago, Illinois, USA; Department of Quantitative Health Sciences, Mayo Clinic Arizona, Scottsdale, Arizona, USA; Melanoma Institute Australia, University of Sydney, Sydney, New South Wales, Australia; Faculty of Medicine and Health, University of Sydney, Sydney, New South Wales, Australia; Department of Melanoma and Surgical Oncology, Royal Prince Alfred Hospital, Sydney, New South Wales, Australia; Department of Surgical Oncology, Erasmus Medical Centre Cancer Institute, Rotterdam, The Netherlands; Department of Public Health, Erasmus University Medical Centre, Rotterdam, The Netherlands; Department of Surgical Oncology, Erasmus Medical Centre Cancer Institute, Rotterdam, The Netherlands; Department of Surgery, Institute of Clinical Sciences, Sahlgrenska Academy, University of Gothenburg, Gothenburg, Sweden; Department of Surgery, Sahlgrenska University Hospital, Gothenburg, Sweden

## Abstract

**Background:**

Recently, a model to predict 5-year recurrence-free survival (RFS) and melanoma-specific survival (MSS) after sentinel lymph node biopsy (SLNB) was published. The aim of this study was to validate that model in a large independent international cohort.

**Methods:**

The database of the Sentinel Lymph Node Working Group (SLNWG) was analysed for patients with malignant melanoma who underwent SLNB. Patients with clinical stage III melanoma, a history of other malignancies, or receiving concomitant systemic therapies during follow-up were excluded. The model’s predictive performance was evaluated using discrimination and calibration metrics in the eligible cohort. Decision curve analysis was performed to assess the clinical value of the model.

**Results:**

The external validation cohort consisted of 6174 patients of the SLNWG from the USA, Europe, and Israel. A positive sentinel node was found in 788 patients (12.8%). The area under the time-dependent receiver operating characteristic (ROC) curve of the external validation was 0.76 (95% c.i. 0.74 to 0.77) for RFS and 0.79 (95% c.i. 0.76 to 0.81) for MSS. The model was well calibrated, as the observed 5-year survival rates aligned closely with the predicted survival rates (calibration slope of 0.98 for RFS and calibration slope of 0.99 for MSS). The model provided a net benefit *versus* the ‘treat all’ and ‘treat none’ strategies at the predetermined probability threshold for recurrence of 45%.

**Conclusion:**

The model demonstrated good performance in a large heterogeneous independent cohort, emphasizing its robustness. Decision curve analysis revealed a clear net benefit of the model over a treat all strategy, highlighting its potential for clinical use.

## Teaser

This study sought to externally validate a model predicting 5-year recurrence-free and melanoma-specific survival. In a large independent cohort consisting of patients from the USA, Europe, and Israel, the model showed good performance, emphasizing its generalizability and robustness. Additionally, decision curve analysis showed a benefit of using the model.

## Introduction

Accurate staging is fundamental for the effective management of patients with melanoma. It informs oncologists and patients about likely disease outcomes and, in turn, enables physicians to determine the most appropriate treatment strategy. However, traditional staging systems do not consider some important prognostic variables. To overcome these limitations, predictive models have been developed that incorporate demographic, clinical, and pathology data^1–4^. Integration of these models into clinical practice, alongside traditional staging systems, enhances the precision and personalization of outcome assessments, thereby supporting more informed clinical decision-making in melanoma care.

Recently, a new prediction model, hereafter called the Predicting REcurrence-Free mElanoma-specific suRvival for patients who underwent a Sentinel node procedure (PREFERS) model, was developed. By using six readily available prognostic variables, this model predicts 5-year recurrence-free survival (RFS) and 5-year melanoma-specific survival (MSS) after sentinel lymph node biopsy (SLNB). With area under the time-dependent receiver operating characteristic (ROC) curve (AUC) values of 0.80 for RFS and 0.81 for MS, PREFERS provides physicians with valuable risk estimates before adjuvant therapy^3^. These individualized risk estimates, combined with the HRs associated with adjuvant therapy, enable discussions focused on absolute risk reductions rather than relative risk reductions, offering a clearer understanding of potential treatment benefits. PREFERS sought to improve upon existing models by including both patients with and without sentinel lymph node (SLN) metastases, as well as accounting for the tumour burden of the largest SLN metastasis^1,2,5^. It demonstrated good performance when externally validated in an independent Australian cohort^3^. Further validation in other large heterogenic cohorts is essential to evaluate the robustness and applicability of the PREFERS model^6^.

The aim of this study was to externally validate the previously published prediction model in a large independent international database.

## Methods

In total, 13 685 patients who underwent SLNB between 1992 and 2018 were assessed for eligibility. Patients were treated in 1 of 15 major cancer centres attached to the Sentinel Lymph Node Working Group (SLNWG). Patients with microsatellites, in-transit, nodal, or any other overt metastases at diagnosis or a history of another malignancy were excluded. Patients who received concomitant systemic therapies were also excluded, as well as patients with missing or discrepant follow-up data (for example unknown last date of follow-up, recurrence before SLNB, or SLNB before date of birth). The study was approved and performed in accordance with the guidelines of the local ethics committee, that is the Erasmus Medical Centre Ethics Committee (MEC2017-375).

### Diagnosis and staging

The study was conducted according to the Transparent Reporting of a multivariable prediction model for Individual Prognosis Or Diagnosis (TRIPOD) guidelines. Surgical procedures were performed similarly to those in the development of the model. Primary tumours were resected with appropriate margins based on national guidelines and SLNB was performed according to techniques published in previous reports^3^. For the present validation study, all patients were retrospectively staged according to the eighth edition of the AJCC staging manual^7^. Follow-up strategies and outcome reporting were at the discretion of the participating institution in accordance with national treatment guidelines for melanoma^8^.

### Data collection

The following clinical data were collected: patient characteristics (age at the time of melanoma diagnosis, sex, and history of other cancers), date of melanoma diagnosis, primary tumour characteristics (location, Breslow thickness, ulceration, histological subtype, and microsatellitosis), date of SLNB, SLN characteristics (tumour burden and microanatomic location of the metastasis (Dewar criteria)), and follow-up data, including data relating to treatments (surgical, systemic, or radiation), disease recurrence, and survival. In contrast to the development data, tumour burden was only available as a categorical variable (<0.1, 0.1–1, and >1 mm) in the validation data. Given that a continuous variable was required in the PREFERS model, the mean tumour burden values from the development data were imputed for each category (0.05, 0.51, and 3.93 mm respectively).

### Outcomes

Outcomes for 5-year probabilities were reported similarly to the previous development of the PREFERS model; the composite outcome was defined as patients experiencing recurrence or all-cause death^3^. Melanoma-specific mortality was defined as death due to melanoma. To align with international nomenclature, RFS and MSS were described as outcomes, and calculated as 1 − (composite outcome) and 1 − (melanoma-specific mortality). RFS was defined as the time from SLNB to the first recurrence of melanoma or death from any cause, whichever occurred first. MSS was defined as the time from SLNB to death due to melanoma.

### Statistical analysis

A sample size calculation was not performed, as all participants who met the inclusion criteria during the interval of interest for analysis were included in the study. Estimates of RFS and MSS unadjusted for case mix were obtained using Kaplan–Meier curves. Median follow-up time was determined using the reverse Kaplan–Meier method. For RFS, patients lost to follow-up were censored, and, for melanoma-specific mortality, those who were lost to follow-up or who died from other or unclear causes were censored. Multivariable imputation by chained equations (using the ‘mice’ package in R) was used for multiple imputation of missing predictor values. To optimize imputation outcomes, the use of variables used for imputation was extended beyond those necessary for the model. Further specifications of the prediction model have previously been described^3^.

### Model performance

Performance of the PREFERS model was assessed both in the full cohort and separately in the countries where the data originated from. Performance was evaluated using calibration and discrimination. Calibration was assessed by plotting the predicted probabilities *versus* the observed frequency of the composite outcome and melanoma-specific mortality. Discrimination was assessed using Harrell’s C-index, Uno’s C-index, and the AUC^9–11^.

### Decision curve analysis (DCA)

DCA was used to evaluate the net benefit for the PREFERS model in comparison with treating all patients or treating no patients at all^6,12^. Net benefit for RFS was calculated by the number of patients correctly predicted to have the composite outcome (true positives) minus the weighted number of patients incorrectly predicted to have the composite outcome (false positives) across a range of threshold probabilities^13^. True positivity and false positivity were defined by setting a probability threshold. The probability threshold reflects a clinical trade-off, balancing the risk of undertreatment against the risk of overtreatment (for example the significance of failing to administer adjuvant therapy to patients who may benefit from it *versus* administering adjuvant therapy and potentially exposing patients to adverse events when the risk of recurrence is low). In this study, a hypothetical probability threshold was used for the composite outcome. The threshold was set at 45% (1 − (mean 5-year RFS))^14^ and reflects the mean risk of the composite outcome for patients with stage IIB melanoma, the minimum requirement for systemic therapy for patients with high-risk stage IIB/C melanoma^14–17^. Additionally, a sensitivity analysis was performed using a probability threshold of 44% (mean RFS of stage IIIA).

All statistical analyses were performed using R (R Foundation for Statistical Computing, Vienna Austria; version 4.1.0) and are available at https://github.com/CHMMaas/ValidateMelanoma.

## Results

### Patient characteristics

**Fig. 1:**
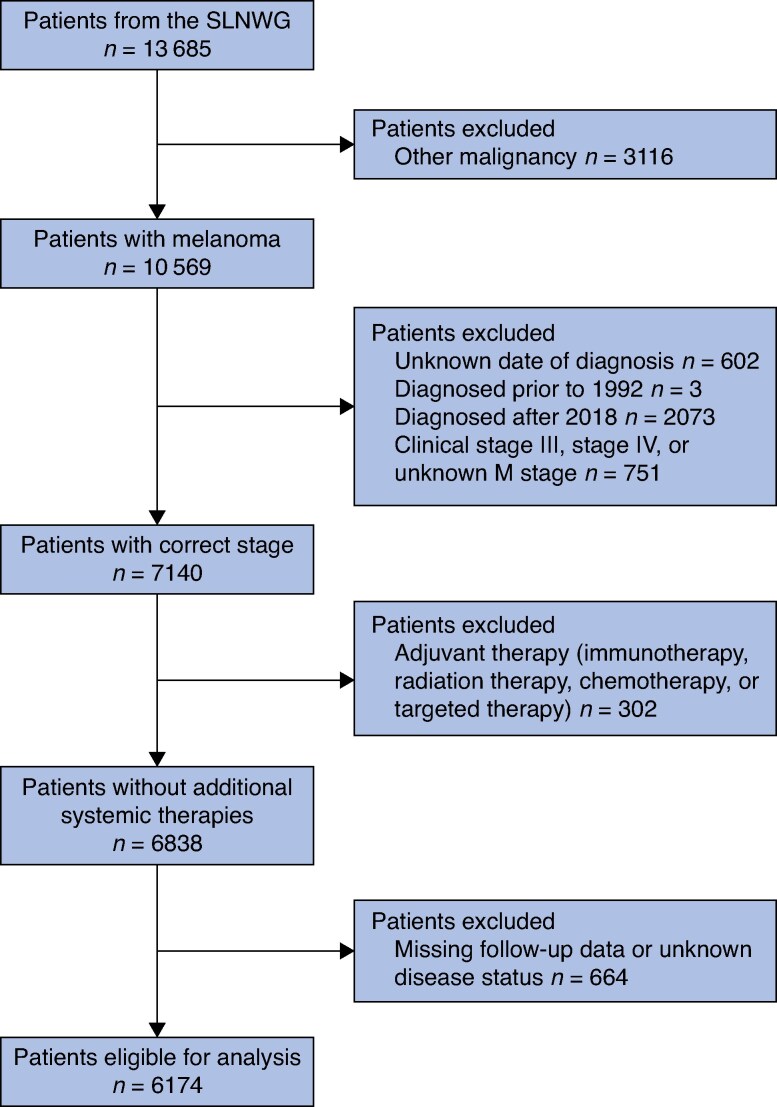
Flow chart for study SLNWG, Sentinel Lymph Node Working Group.

A total of 13 685 patients from the SLNWG were assessed for study eligibility, of whom 6174 were included (*Fig. 1*). Of these 6174 patients, 4572 patients (74.1%) originated from the USA, 974 patients (15.8%) originated from Sweden, 389 patients (6.3%) originated from Israel, 237 patients (3.8%) originated from Italy, and 2 patients originated from the Netherlands (*Table 1*). Respectively, 2734 (44.3%) and 3438 (55.7%) females and males were included. The mean(s.d.) age at the time of SLNB was 56.5(15.9) years (*Table 1*).

**Table 1:** Descriptive statistics

	Total* (*n* = 6174)	USA (*n* = 4572)	Sweden (*n* = 974)	Israel (*n* = 389)	Italy (*n* = 237)
**SLN status**					
Negative	5343 (86.5)	3966 (86.7)	829 (85.1)	345 (88.7)	202 (85.2)
Positive	788 (12.8)	582 (12.7)	140 (14.4)	30 (7.7)	35 (14.8)
Missing	43 (0.7)	24 (0.5)	5 (0.5)	14 (3.6)	0 (0.0)
**Sex**					
Male	3438 (55.7)	2620 (57.3)	494 (50.7)	202 (51.9)	121 (51.1)
Female	2734 (44.3)	1950 (42.7)	480 (49.3)	187 (48.1)	116 (48.9)
Missing	2 (0.0)	2 (0.0)	0 (0.0)	0 (0.0)	0 (0.0)
**Age at time of SLNB (years)**					
Mean(s.d.)	56.5(15.9)	56.3(16.0)	58.5(15.1)	56.0(14.8)	53.1(16.2)
Minimum—maximum	2.0–97.0	2.0–97.0	16.0–90.0	17.0–89.0	18.0–88.0
**Ulceration**					
No	4050 (65.6)	3027 (66.2)	610 (62.7)	364 (93.6)	128 (54.0)
Yes	1423 (23.0)	912 (19.9)	315 (32.3)	89 (22.9)	107 (45.2)
Missing	701 (11.4)	633 (13.9)	49 (5.0)	17 (4.4)	2 (0.8)
**Location**					
Arm	1285 (20.8)	1011 (22.1)	162 (16.6)	80 (20.6)	32 (13.5)
Head and neck	965 (15.6)	887 (19.4)	8 (0.8)	56 (14.4)	14 (5.9)
Leg	1479 (24.0)	1003 (21.9)	297 (30.5)	118 (30.3)	61 (25.7)
Trunk	2433 (39.4)	1660 (36.3)	507 (52.1)	135 (34.7)	129 (54.4)
Missing	12 (0.2)	11 (0.2)	0 (0.0)	0 (0.0)	1 (0.4)
**Histology**					
Acral	204 (3.3)	171 (3.7)	28 (2.9)	2 (0.5)	3 (1.3)
Desmoplastic	119 (1.9)	105 (2.3)	12 (1.2)	2 (0.5)	0 (0.0)
Lentigo maligna	98 (1.6)	82 (1.8)	12 (1.2)	2 (0.5)	2 (0.8)
Nevoid melanoma	63 (1.0)	30 (0.7)	28 (2.9)	4 (1.0)	1 (0.4)
Nodular	1040 (16.8)	562 (12.3)	270 (27.7)	58 (14.9)	150 (63.3)
Spindle	59 (1.0)	55 (1.2)	3 (0.3)	1 (0.3)	0 (0.0)
Superficial spreading	1813 (29.4)	1337 (29.2)	386 (39.6)	32 (8.2)	56 (23.6)
Other^†^	926 (15.0)	793 (17.3)	97 (10.0)	12 (3.1)	24 (10.1)
Missing	1852 (30.0)	1437 (31.4)	138 (14.2)	276 (71.0)	1 (0.4)
**Breslow thickness (mm)**					
Median (interquartile range)	1.4 (1.0–2.5)	1.4 (0.9–2.4)	1.8 (1.2–3.0)	1.3 (0.9–2.5)	1.8 (1.1–3.2)
Minimum—maximum	0.1–30.0	0.1–30.0	0.2–17.0	0.3–13.0	0.3–30.0
Missing	57	43	1	11	2
**Mitosis**					
<1/mm^2^	581 (9.4)	536 (11.7)	6 (0.6)	9 (2.3)	30 (12.7)
Missing	3034 (49.1)	1763 (38.6)	932 (95.7)	295 (75.8)	44 (18.6)
**SLN tumour burden^‡^**					
<0.1 mm	14 (1.8)	6 (1.0)	8 (5.7)	0 (0.0)	0 (0.0)
0.1–1.0 mm	62 (7.9)	24 (4.1)	36 (25.7)	2 (6.7)	0 (0.0)
>1.0 mm	73 (9.3)	21 (3.6)	50 (35.7)	0 (0.0)	1 (2.9)
Missing	639 (81.1)	531 (91.2)	46 (32.9)	28 (93.3)	34 (97.1)
**Dewar^‡^**					
Combined	31 (3.9)	11 (1.9)	11 (7.9)	0 (0.0)	9 (25.7)
Extensive	12 (1.5)	1 (0.2)	11 (7.9)	0 (0.0)	0 (0.0)
Multifocal	28 (3.6)	5 (0.9)	23 (16.4)	0 (0.0)	0 (0.0)
Parenchymal	22 (2.8)	0 (0.0)	19 (13.6)	1 (3.3)	2 (5.7)
Subcapsular	98 (12.4)	50 (8.6)	32 (22.9)	0 (0.0)	16 (45.7)
Missing	597 (75.8)	515 (88.5)	44 (31.4)	29 (96.7)	8 (22.9)
Duration of follow-up (years), median (interquartile range)	5.9 (2.1–9.9)	5.7 (2.0–9.4)	9.7 (5.8–14.2)	4.9 (1.6–9.1)	0.6 (0.2–0.9)

Values are *n* (%) unless otherwise indicated. Age at time of SLNB was normally distributed and Breslow thickness was not normally distributed. *Total includes 4572 patients who originated from the USA, 974 patients who originated from Sweden, 389 patients who originated from Israel, 237 patients who originated from Italy, and 2 patients who originated from the Netherlands. ^†^Other histology types include melanoma of childhood, persistent melanoma, melanoma arising from blue nevus, melanoma arising from giant congenital nevus, and neurotropic melanoma. ^‡^SLN tumour burden and Dewar were only computed for the patients with a positive SLN status (788 patients). SLN, sentinel lymph node; SLNB, sentinel lymph node biopsy.

### Survival outcomes

The median duration of follow-up was 5.9 (interquartile range 2.1–9.9) years (*Table 1*). Of 6174 patients, the composite outcome occurred in 1158 patients (19%) within 5 years (*Table 2* and *Fig. S1a*,*b*). The 5-year RFS rate for the overall cohort was 75.4% (95% c.i. 74.2% to 76.7%). For patients with a positive SLN this was 50.1% (95% c.i. 46.4% to 54.1%) and for patients with a negative SLN this was 79.4% (95% c.i. 78.1% to 80.7%). In patients with a positive SLN, 151 patients (19%) died due to melanoma in the first 5 years after SLNB; in contrast, in patients with a negative SLN, 246 patients (5%) died due to melanoma in the first 5 years after SLNB. Overall, the 5-year MSS was 90.7% (95% c.i. 89.8% to 91.6%), being 75.1% (95% c.i. 71.7% to 78.7%) for patients with a positive SLN and 93.1% (95% c.i. 92.3% to 94.0%) for patients with a negative SLN (*Table 2*).

**Table 2:** Composite outcome or deaths due to melanoma within 5 years after SLNB and 5-year Kaplan–Meier estimates of RFS and MSS in the validation cohort

	Recurrence or death	5-year RFS (95% c.i.), %	Melanoma-specific deaths	5-year MSS (95% c.i.), %
**Full cohort**				
All (*n* = 6174)	1158 (19)	75.4 (74.2,76.7)	399 (6)	90.7 (89.8,91.6)
Positive SLN (*n* = 788)	336 (43)	50.1 (46.4,54.1)	151 (19)	75.1 (71.7,78.7)
Negative SLN (*n* = 5343)	818 (15)	79.4 (78.1,80.7)	246 (5)	93.1 (92.3,94.0)
**USA**				
All (*n* = 4572)	878 (19)	74.5 (73.0,76.0)	279 (6)	91.0 (90.0,92.0)
Positive SLN (*n* = 582)	239 (41)	51.9 (47.5,56.6)	108 (19)	75.9 (71.9,80.0)
Negative SLN (*n* = 3966)	635 (16)	78.1 (76.6,79.7)	169 (4)	93.5 (92.5,94.4)
**Sweden**				
All (*n* = 974)	224 (23)	76.5 (73.8,79.2)	108 (11)	88.4 (86.3,90.5)
Positive SLN (*n* = 140)	75 (54)	45.7 (38.1,54.9)	41 (29)	69.7 (62.4,78.0)
Negative SLN (*n* = 829)	149 (18)	81.5 (78.9,84.3)	67 (8)	91.4 (89.5,93.4)
**Israel**				
All (*n* = 389)	45 (12)	84.3 (80.1,88.7)	10 (3)	96.0 (93.6,98.5)
Positive SLN (*n* = 30)	12 (40)	54.6 (38.4,77.6)	0 (0)	100.0 (100.0,100.0)
Negative SLN (*n* = 345)	33 (10)	86.6 (82.4,91.1)	10 (3)	95.5 (92.8,98.3)

Values are *n* (%) unless otherwise indicated. SLNB, sentinel lymph node biopsy; RFS, recurrence-free survival; MSS, melanoma-specific survival; SLN, sentinel lymph node.

### External validation

**Fig. 2:**
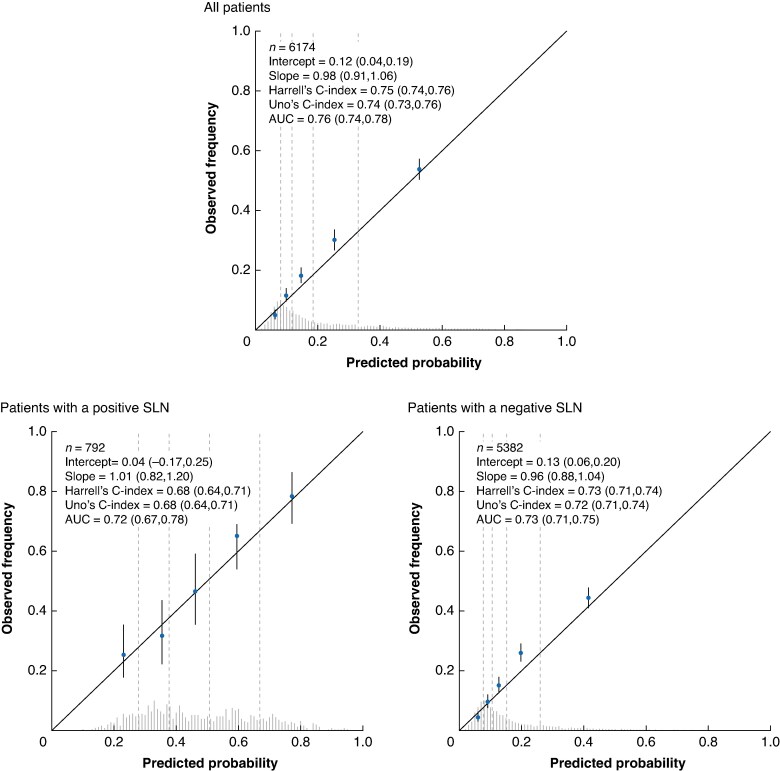
Calibration and discrimination of the model predicting RFS for all patients, only patients with a positive SLN, and only patients with a negative SLN RFS, recurrence-free survival; SLN, sentinel lymph node. Values in parentheses are 95% confidence intervals.

**Fig. 3:**
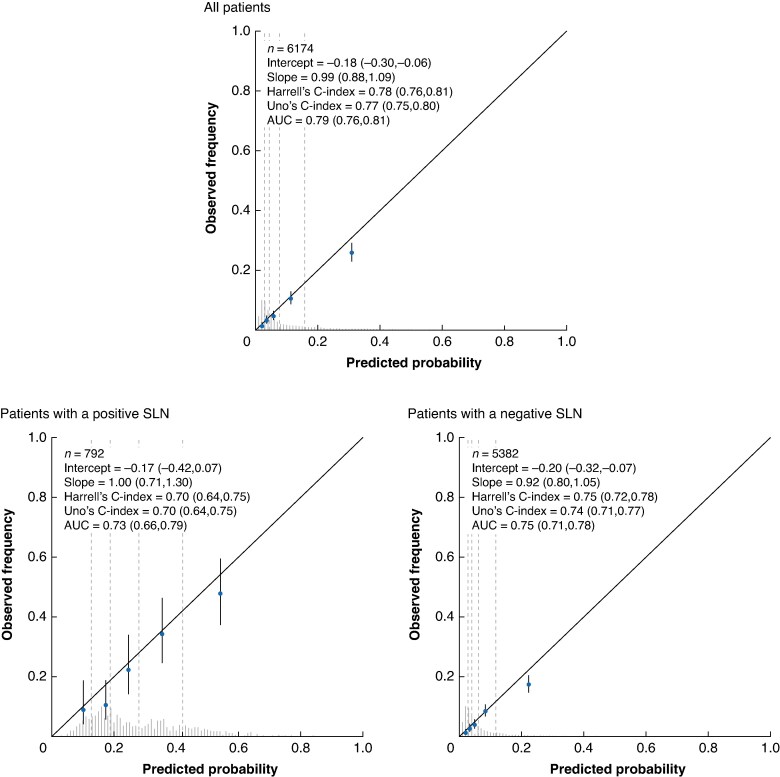
Calibration and discrimination of the model predicting MSS for all patients, only patients with a positive SLN, and only patients with a negative SLN MSS, melanoma-specific survival; SLN, sentinel lymph node. Values in parentheses are 95% confidence intervals.

For the full cohort, the AUC of the external validation was 0.76 (95% c.i. 0.74 to 0.77) for RFS and 0.79 (95% c.i. 0.76 to 0.81) for MSS. The prognostic model appeared to be well calibrated, as the observed 5-year survival rates aligned closely with the predicted 5-year survival rates (calibration slope of 0.98 for RFS (*Fig. 2*) and calibration slope of 0.99 for MSS (*Fig. 3*)). Across the different geographical locations, the model was well calibrated, with only slight underestimation and overestimation of risk (*Fig. S2a*,*b*). Due to limited follow-up time, separate assessment of performance was not conducted for the Italian data.

### DCA

Providing adjuvant therapy to all patients (that is a treat all strategy) would have resulted in 1519 patients receiving adjuvant treatment who would experience the composite outcome, while also treating 4655 patients who would not experience the outcome. Meanwhile, a treat none strategy, yielded no net benefit, as none of the patients would have been incorrectly selected for adjuvant therapy and none would have been incorrectly passed for adjuvant therapy. The net benefit of such a strategy is therefore, by definition, zero.

**Fig. 4:**
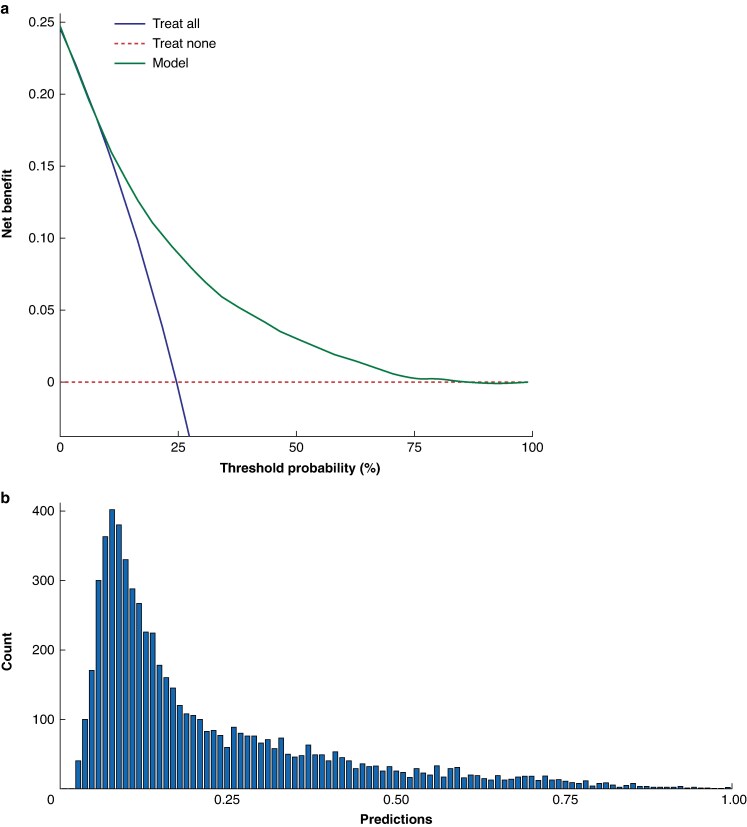
Decision curve analysis for the composite outcome of recurrence or death for the full validation cohort of patients who underwent SLNB in the USA, Sweden, Israel, Italy, and the Netherlands **a** The *y*-axis represents the net benefit at each probability threshold, reflecting the proportion of patients with the composite outcome correctly identified by the treatment strategy, adjusted for the penalty of incorrectly identifying patients without the composite outcome. The *x*-axis represents the continuum of threshold probabilities for experiencing the composite outcome within 5 years. The green line represents the net benefit of treating no individuals with adjuvant therapy (treat none approach). The red line represents the net benefit of treating all individuals with adjuvant therapy (treat all approach). The blue line represents the net benefit of treating patients according to the individualized predictions of experiencing the composite outcome resulting from the PREFERS model across the continuum of threshold probabilities (model approach). **b** Displays the distribution of predicted probabilities for the composite outcome of recurrence or death. SLNB, sentinel lymph node biopsy; PREFERS, Predicting REcurrence-Free mElanoma-specific suRvival for patients who underwent a Sentinel node procedure.

The PREFERS model provided higher net benefit *versus* the treat all strategy at the probability threshold of 45%. At this threshold, the model accurately predicted the composite outcome in 465 patients (true positives) and incorrectly predicted the composite outcome in 274 patients (false positives; *Table 3*), which resulted in a net benefit of 0.04. Adjuvant therapy for all patients at this probability threshold resulted in a net benefit of −0.37 (*Table 3* and *Fig. 4*). Using the model would have resulted in 5422 patients (87.8%) not receiving adjuvant immunotherapy, though 1037 of these 5422 patients would experience the composite outcome. The sensitivity analysis using a probability threshold of 44% yielded similar results as the threshold of 45%.

**Table 3:** Classification and utility measures of the prediction model at several probability thresholds

Probability threshold, %	Sensitivity, %	Specificity, %	True positives	False positives	True negatives	False negatives	PPV, %	NPV, %	Net benefit of the model	Net benefit of a treat all strategy	Net benefit of a treat none strategy
44	9.8	78.4	477	287	1042	4368	62.5	19.3	0.04	−0.35	0.00
45	9.6	79.4	465	274	1054	4381	62.9	19.4	0.04	−0.37	0.00
50	8.0	85.3	385	196	1134	4459	66.3	20.3	0.03	−0.51	0.00
55	6.6	89.1	318	147	1201	4508	68.4	21.0	0.02	−0.68	0.00
60	5.1	93.0	243	96	1276	4559	71.8	21.9	0.02	−0.88	0.00

Values are *n* unless otherwise indicated. PPV, positive predictive value; NPV, negative predictive value.

## Discussion

Utilizing a large independent international cohort, this study sought to validate the previously developed PREFERS model to predict RFS and MSS in patients after SLNB. While initial external validation in an Australian cohort demonstrated good generalizability, further validation in other diverse populations was necessary to assess the robustness and clinical value of the PREFERS model. In this validation, the model showed a predictive performance that was slightly lower than, yet comparable to, that of the original development study.

According to the literature, risk prediction models often demonstrate reduced performance during external validation compared with during their initial development^18^. This is a consequence of variations in populations, variations in measurement of outcomes, and changes over time in both factors. However, these variations, when accounted for properly, can improve the robustness and generalizability of a model. To achieve meaningful validation, it is therefore important to test models in heterogeneous data sets that are representative of the intended target population. However, it is equally important to recognize that a prediction model is never fully validated. This is because predictive performance may vary across different settings and change over time due to changes in population characteristics following advances in healthcare management. For example, given that healthcare systems vary significantly between countries, regions, and hospital types (for example academic *versus* community), a model based on patients from an academic hospital in the USA may not perform equally well in a cohort from a community hospital in the Netherlands^19^. As the PREFERS model was developed in hospitals in different countries around Europe, the targeted population was partially accounted for during its creation. This subsequent validation in a diverse cohort from hospitals in the USA, Europe, and Israel confirmed its robustness across varied clinical settings.

It is a common interpretation error for investigators to choose an ideal probability threshold based on the DCA, as this reverses the relationship between threshold probability and evaluation of a model. Instead, it is crucial for investigators to first select a clinically relevant threshold probability by considering the relative harms of avoiding intervention for a patient with disease *versus* unnecessarily intervening for a patient who is disease free^13^. Unlike thresholds for other interventions, such as the 5% threshold probability for considering SLNB^20^, no minimum probability threshold has been defined for considering adjuvant therapy based on 5-year RFS. Consequently, the mean risk for recurrence or death (45%) was used for patients with stage IIB as the reference threshold^14^. At this probability threshold, the model demonstrated a net benefit of 0.04, which indicates that the model provides a small positive benefit when it comes to deciding who should receive treatment or an intervention. This indicates that using this model is slightly better than not having a model at all. DCA further shows that the use of the model yields a higher net benefit than a strategy where all patients would receive treatment (net benefit −0.37). The negative net benefit of the treat all strategy at this threshold suggests that treating everyone would do more harm than good, as it involves treating many patients who would not experience recurrence within 5 years. This highlights the potential of the model in guiding treatment decisions and reducing the risks associated with overtreating all patients or undertreating high-risk individuals, supporting a more personalized and efficient treatment strategy. Although the original model included MSS as a primary outcome, current clinical practice bases treatment decisions on RFS rather than MSS. Therefore, MSS-based DCA was not included as a primary outcome in this study; instead, it is provided as supplementary material (Fig. S3).

The strength of this study lies in the quality of its data set. For the development of the PREFERS model, a cohort of patients who underwent SLNB between 1997 and 2013 was specifically chosen to minimize potential treatment bias introduced by adjuvant therapies. Although only a very small percentage of patients received adjuvant therapies after recurrence, it is possible that some patients were not accurately recorded in the data set. However, in the SLNWG data, additional treatments were well registered, allowing careful exclusion of patients who received (neo)adjuvant therapies. This made the final cohort suited for the appropriate target population. Furthermore, the SLNWG cohort, sourced from 15 major cancer centres divided over different continents, with each hospital following diverse melanoma treatment and follow-up guidelines, provided a highly heterogeneous and representative population, ideal for external validation.

This study also has certain limitations. Although the international scope of the database enhances its diversity, variations in surgical and pathological guidelines in different countries increase the risk for missing data. Data well registered in one country may be considered less important in another due to differing clinical practices and protocols. In the SLNWG data set, SLN tumour burden, one of the variables required for the PREFERS model, was missing in the majority of patients. This may have influenced the discriminative performance, as reflected by a slightly lower C-index in the external validation cohort compared with the development cohort (C-index of 0.76 *versus* C-index of 0.80 respectively). This difference is likely an underestimation due to the substantial amount (∼80%) of missing information concerning SLN tumour burden. Although tumour burden is a well-established prognostic factor in patients with SLN metastases^21,22^, its variable importance during model development was relatively low (Wald test χ^2^ = 20)^3^. This likely explains why the effect on the predicted outcome is relatively limited. Furthermore, due to differences in tumour burden distribution, some degree of miscalibration may have occurred. However, assuming the distribution of tumour burden in the external validation cohort is similar to that in the development cohort, substantial miscalibration is unlikely.

## Supplementary Material

znaf037_Supplementary_Data

## Data Availability

Requests for access to the deidentified patient data that form the basis of the authors’ findings should be directed to the Sentinel Lymph Node Working Group (SLNWG) of the Sentinel Node Oncology Foundation. However, access to the data is at the Sentinel Node Oncology Foundation’s discretion.
