# Supplementary material for: External validation of a model to predict recurrence-free and melanoma-specific survival for patients with melanoma after sentinel node biopsy
Source: Br J Surg. 2025 Apr 17;112(4):znaf037. doi: 10.1093/bjs/znaf037 (PMC12004364; doi:10.1093/bjs/znaf037)
Supplement: znaf037_Supplementary_Data [file znaf037_supplementary_data.docx]

**External validation of a model to predict recurrence-free and melanoma-specific survival for patients with melanoma after sentinel node biopsy**

Robert C Stassen^1^, Carolien C H M Maas^2^, Stanley P Leong^3^, Mohammed Kashani-Sabet^3^, Richard L. White Jr.^4^, Barbara A Pockaj^5^, Jonathan S Zager^6^, Schlomo Schneebaum^7,^ John T Vetto^8^, Eli Avisar^9^, J. Harrison Howard^10^, Cristina O´Donoghue^11^, Heidi Kosiorek^12^, Alexander CJ van Akkooi^13-15^, Cornelis Verhoef^1^, David van Klaveren^2^, Dirk J Grünhagen^1^*, Roger Olofsson Bagge^16,17^*

*Shared senior authors

^1^Department of Surgical Oncology, Erasmus Medical Centre Cancer Institute, Rotterdam, Netherlands.

^2^Department of Public Health, Erasmus University Medical Centre, Rotterdam, Netherlands.

^3^Department of Surgery, California Pacific Medical Center and Research Institute, San Francisco, CA.

^4^Levine Cancer Institute, Carolinas Medical Center, Atrium Health, Charlotte, NC.

^5^Department of Surgery, Mayo Clinic, Phoenix, AZ.

^6^Department of Cutaneous Oncology, Moffitt Cancer Center, Tampa, FL.

^7^Department of Surgery, Tel-Aviv Sourasky Medical Center, Israel.

^8^Division of Surgical Oncology, Oregon Health & Science University, Portland, OR.

^9^Sylvester Comprehensive Cancer Center, University of Miami Miller School of Medicine, Miami, FL, USA.

^10^Department of Surgery, University of South Alabama, Mobile, Alabama, U.S.A

^11^Department of Surgery, Rush University Medical Center, Chicago, Illinois, U.S.A.

^12^Department of Quantitative Health Sciences, Mayo Clinic Arizona, Scottsdale, Arizona

^13^Melanoma Institute Australia, University of Sydney, Sydney, NSW, Australia

^14^Faculty of Medicine and Health, University of Sydney, Sydney, NSW, Australia

^15^Department of Melanoma Surgical Oncology, Royal Prince Alfred Hospital, Sydney, NSW, Australia

^16^Department of Surgery, Institute of Clinical Sciences, Sahlgrenska Academy, University of Gothenburg, Gothenburg, Sweden.

^17^Department of Surgery, Sahlgrenska University Hospital, Gothenburg, Sweden.

**Corresponding author.** Robert Stassen, Department of surgical oncology, Erasmus Medical Centre – Cancer Institute, Dr. Molewaterplein 40, 3015 GD, Rotterdam, The Netherlands, Tel: +31107041902, Email: [r.stassen@erasmusmc.nl](mailto:r.stassen@erasmusmc.nl)

**ORCID ID**: 0000-0001-6196-6966

**Supplementary Materials - Index**

| **Supplementary Figures and Tables** |  |
| --- | --- |
| Figure S1a. Kaplan-Meier curve of recurrence-free survival for the validation data. | *pag. 3* |
| Figure S1b. Kaplan-Meier curve of melanoma-specific survival for the validation data. | *pag. 4* |
| Figure S2. Calibration plots of the model predicting 5-year recurrence-free survival for all patients and each country separately in the validation data. | *pag. 5* |
| Figure S2. Calibration plots of the model predicting 5-year melanoma-specific survival for all patients and each country separately in the validation data. | *pag. 6* |
| Figure S3. Decision curve analysis for death due to melanoma on the full validation cohort of patients who underwent SLNB in the United Stated of America, Sweden, Israel, Italy, and Netherlands. | *pag. 7* |

**Supplementary Figures and Tables**

**Supplementary Figure 1a. Kaplan-Meier curve of recurrence-free survival for the validation data.**


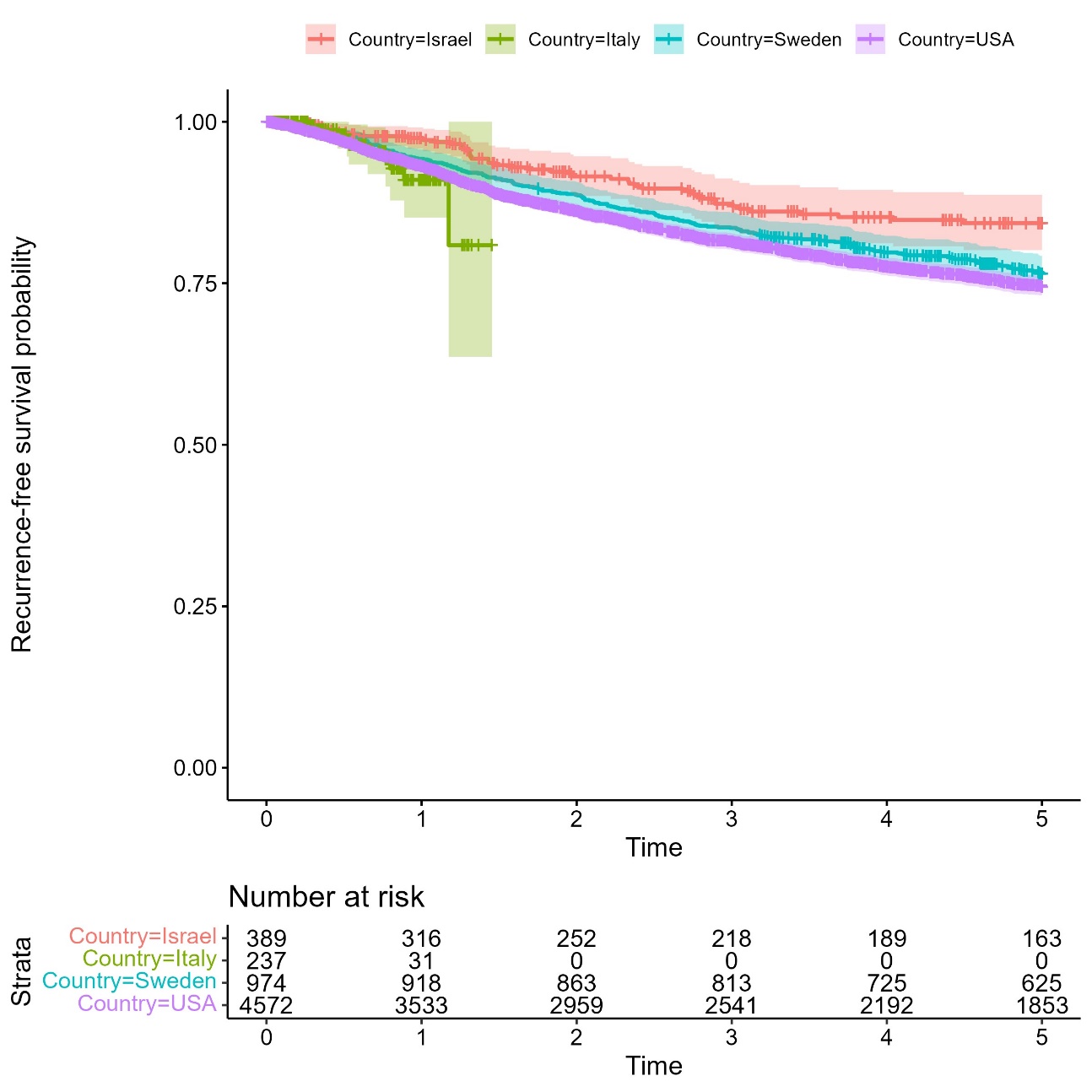


**Supplementary Figure 1b. Kaplan-Meier curve of melanoma-specific survival for the validation data.**


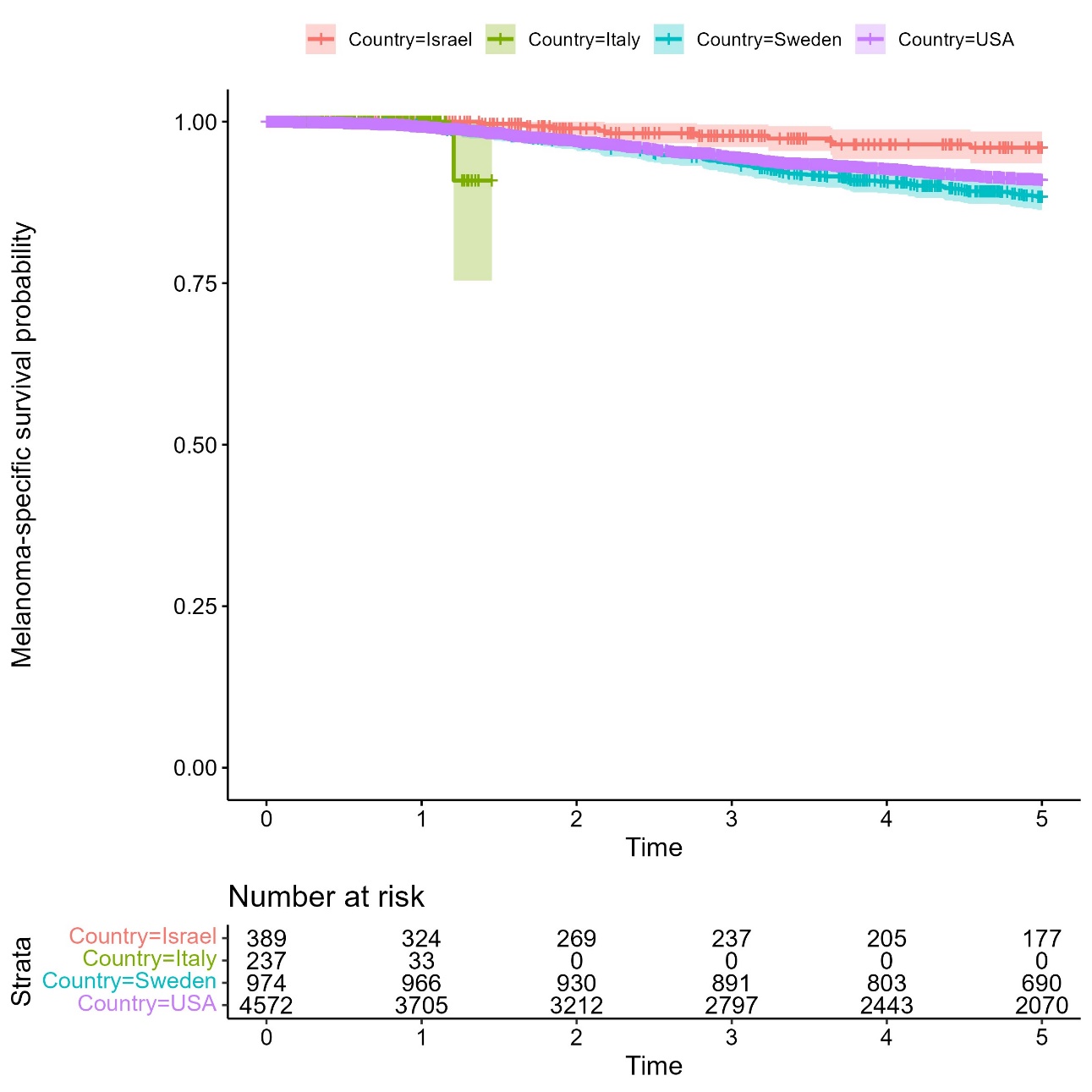


**Supplementary Figure 2a. Calibration plots of the model predicting 5-year RFS for all patients and each country separately in the validation data.**


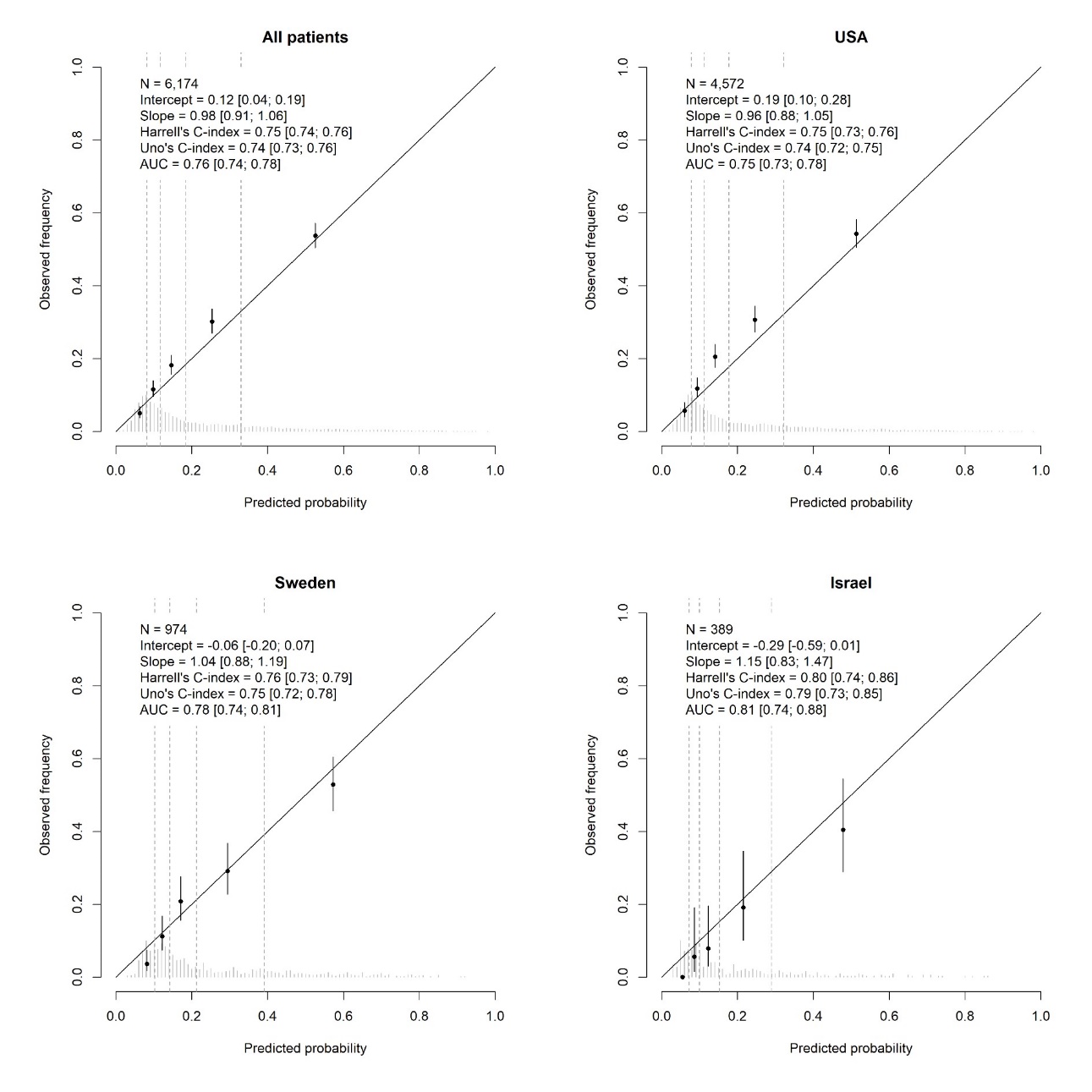


**Supplementary Figure 2b.** **Calibration plots of the model predicting 5-year MSS for all patients and each country separately in the validation data.**


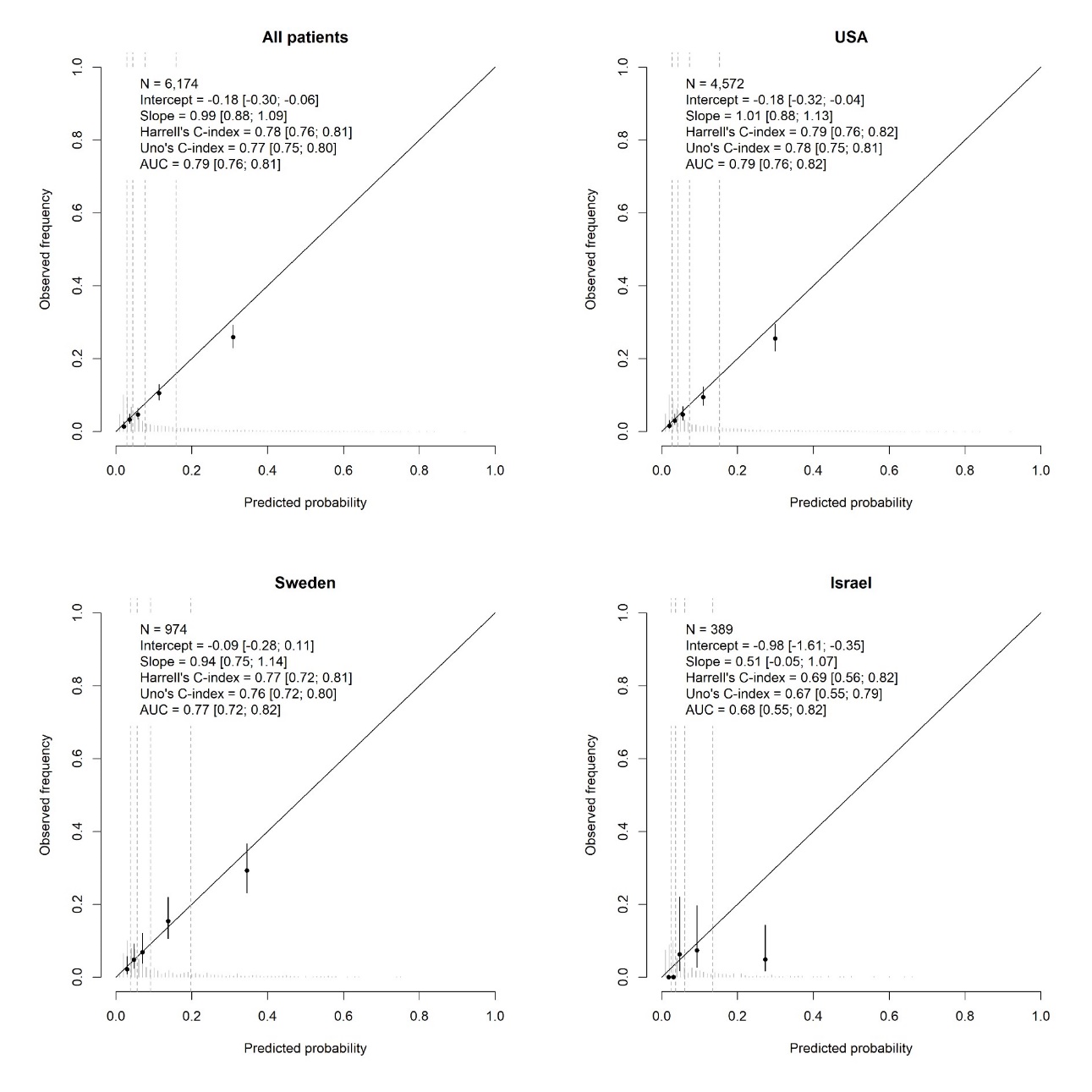


**Supplementary Figure 3.** Decision curve analysis for death due to melanoma on the full validation cohort of patients who underwent SLNB in the United Stated of America, Sweden, Israel, Italy, and Netherlands.


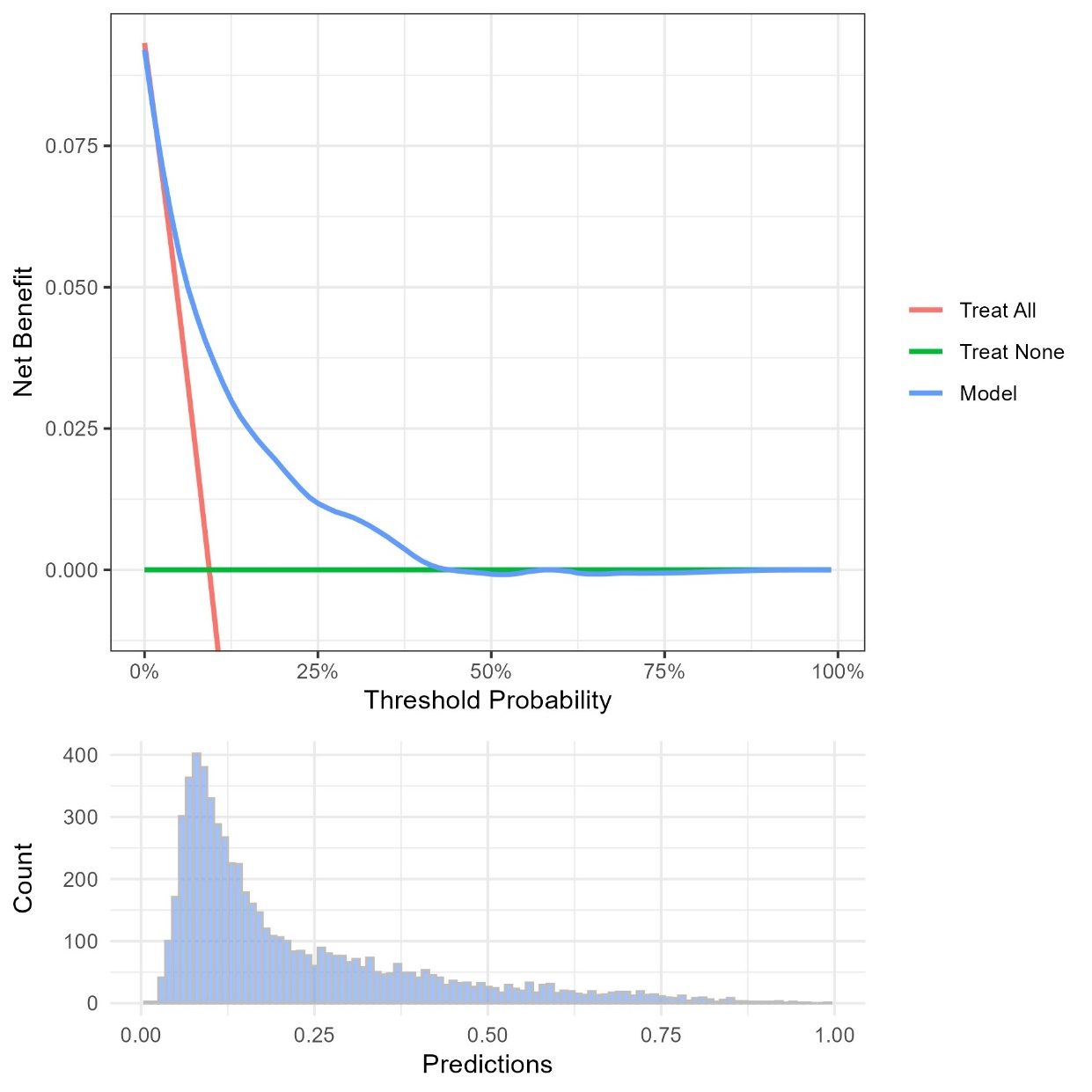


Figure legend:

Top figure: The Y-axis represents the net benefit at each probability threshold, reflecting the proportion of patients with death due to melanoma correctly identified by the treatment strategy, adjusted for the penalty of incorrectly identifying patients without death due to melanoma. The X-axis represents the continuum of threshold probabilities for experiencing death due to melanoma within 5 years. The green line represents net benefit of treating no individuals with adjuvant therapy (‘treat none’ approach). The red line represents net benefit of treating all individuals with adjuvant therapy (‘treat all’ approach). The blue line represents net benefit of treating patients according to the individualized predictions of experiencing the death due to melanoma resulting from the model across the continuous of threshold probabilities (‘model’ approach).

Bottom figure: Displays the distribution of predicted probabilities for melanoma-specific survival
